# Transcriptional Regulation and WGCNA Studies of Leaf Abscission in Cotton Cultivars FU75 and 518-48 Under Chemical Defoliant Treatment

**DOI:** 10.3390/biology15010074

**Published:** 2025-12-31

**Authors:** Rui Yang, Baoguang Xing, Bei Wu, Zhengyang Wang, Wen Zhang, Tao Lu, Fuqiang Zhao, Qingtao Zeng, Yongbo Wang, Pengtao Li

**Affiliations:** 1 Xinjiang Production and Construction Corps Seventh Division Agricultural Research Institute, Kuitun 833200, China; 15388465669@163.com (R.Y.); 18699211120@163.com (Z.W.); zhangwenshzu@163.com (W.Z.); 18139375819@163.com (T.L.); ktzfq@126.com (F.Z.); zy59720@163.com (Q.Z.); 2 Anyang Institute of Technology, Anyang 455000, China; 19937820150@163.com (B.X.); 18734321011@163.com (B.W.); 3 Cotton and Sericulture Research Institute of Hunan Province, Changsha 410127, China

**Keywords:** thidiazuron, cotton, transcriptional regulation, WGCNA, plant hormone signal transduction, MAPK signaling pathway-plant

## Abstract

Leaf abscission is a cell separation process that occurs throughout the entire plant life cycle, leading to the detachment of tissues or organs. The application of chemical defoliants to induce cotton leaf abscission not only saves the energy required for maintaining life processes but also facilitates mechanical harvesting. However, the molecular mechanisms underlying cotton leaf abscission remain poorly understood. In this study, multiple comparative analyses of gene expression differences were conducted between two cotton cultivars with different sensitivities to chemical defoliant Thidiazuron (TDZ) after TDZ application, resulting in 1,505,720,260 clean reads together with the average 92.77% of Q30 base percentage and 43.13% of GC content. A total of 35,739 differentially expressed genes (DEGs) were identified and these DEGs were mainly enriched in pathways of zeatin biosynthesis, secondary metabolite biosynthesis, and hormone metabolic processes. Integration of temporal expression pattern analysis and weighted gene co-expression network analysis (WGCNA) revealed that plant hormone signal transduction and MAPK signaling pathways might play important roles in TDZ-induced leaf abscission. Among them, a *sucrose non-fermenting 1 (SNF1)-related protein kinase 2* gene (*SnRK2*, *GH_A11G1981* and *GH_D11G2017*) in the abscisic acid (ABA) signaling pathway might be a potential key regulatory factor in defoliant induced leaf abscission. These findings provide novel insights into understanding the molecular mechanisms of chemical defoliant-induced leaf abscission in cotton and lay a foundation for future breeding programs and practical applications in cotton production.

## Simple Summary

In cotton production, defoliation treatment using chemicals before picking is a conventional cultivation measurement for mechanical harvesting. However, the mechanism by which defoliants induce cotton leaf shedding still remains not fully understood. After treating the defoliant thidiazuron (TDZ) on its sensitive variety FU75 and insensitive variety 518-48, this study found that phytohormone signal transduction and MAPK signaling pathway might play important roles in TDZ-induced leaf abscission. Further analysis of the DEGs using Short Time-series Expression Miner (STEM) and Weighted Gene Co-expression Network Analysis (WGCNA) revealed that *sucrose non-fermenting 1 (SNF1)-related protein kinase 2* (*SnRK2*) is a key candidate gene in the abscisic acid (ABA) pathway. These findings provide new insights into the molecular mechanism of chemical defoliant-induced cotton leaf abscission and lay a foundation for subsequent research and potential applications in cotton production.

## 1. Introduction

Abscission refers to the natural detachment of plant organs or tissues, typically leaves, flowers, fruits or seeds, from the main stems [1]. This biological process is triggered by various external environmental factors, including temperature changes, water stress, and mineral nutrient deficiencies, and physiological conditions, including fading, senescence, maturation, or hormonal regulation. Organ abscission occurs throughout the plant life cycle and represents a self-pruning mechanism that balances resource allocation and environmental adaptation, thereby avoiding wasting energy required for sustaining life. For instance, under drought stress, plants might shed their leaves to reduce water loss through reducing transpiration. Plants may also shed some of their excessive flowers or young fruits to allow more energy allocated to the remaining ones. The abscission process makes critical contributions on agriculture production. For example, in cereal crops, seed shattering, a form of abscission, is a key factor limiting yield before harvest [2]. Conversely, in cotton, leaf abscission facilitates mechanical harvesting and reduces picking costs [3,4]. In sugarcane, leaf abscission increases field space, improves ventilation, light penetration, and lodging resistance, while reduces the spread of pests and diseases, ultimately enhances yield [5,6,7]. The economic impact of leaf shedding is dual in nature, as it may lead to certain losses while simultaneously offering opportunities for active utilization and conversion into valuable resources. This approach entails leveraging technology to modulate the leaf-shedding process, thereby enhancing agricultural efficiency. A prominent example is the application of chemical defoliants—such as ethephon—prior to mechanized cotton harvesting, which induces synchronized leaf abscission, minimizes contamination during harvest, and significantly improves fiber quality.

The regions where plant organ abscission occurs is known as the abscission zones (AZs), characterized by small, densely packed cytoplasmic cells interconnected by plasmodesmata [8]. The process of plant organ abscission could be divided into four stages: establishment of the AZs, activation of abscission signaling, cell separation, and post-abscission protective layer differentiation [9]. Previous researches have shown that plant organ abscission involves multiple signaling pathways and is controlled by a number of genes. In litchi, the gene *LcKNAT1* has been identified to negatively regulate abscission of young fruits by inhibiting ethylene biosynthesis [10]. In tomato, the MADS-box gene *JOINTLESS* is associated with floral organ development, and its deletion leads to the failure of AZ activation in pedicel [11]. In Arabidopsis, the FOREVER YOUNG FLOWER (FYF) like gene has been validated to inhibit floral abscission and senescence [12], while in *Phalaenopsis*, silencing of *PaFYF1/2* promotes flower senescence and abscission [13]. In Arabidopsis, Floral organ abscission in *Arabidopsis* is also regulated by a specific signaling peptide INFLORESCENCE DEFICIENT IN ABSCISSION (IDA) and a receptor complex consisting of the receptor-like kinase HAESA and HAESA-LIKE2, which activates the mitogen-activated protein kinase (MAPK) cascade to promote AZ cell establishment [14]. Plant hormones also play a crucial role in regulating organ abscission [15]. For instance, in poplar, auxin acts as a long-distance signal that functions independently of ethylene signaling in leaf abscission [16]. In lupine, abscisic acid (ABA) produced from rot caps and withered leavse in plants and ethylene alter the protein abundance of lipoxygenase (LOX) and the accumulation of jasmonates in AZ cells, up-regulating floral organ abscission [17]. In grapevine, *VlbZIP14* directly activates *VlCOMT* by binding to the G-box motif in its promoter, promoting lignin biosynthesis and thereby inhibiting fruit abscission [18].

Transcriptomics is a discipline that studies the gene expression profiles and transcriptional regulatory mechanisms of cells or tissues under specific developmental stages or physiological conditions, serving as a tool of dissecting the regulatory mechanisms of cellular phenotypes and properties [19,20]. In a previous study, via comparing the apple samples treated with hexanal and control, a total of 726 differentially expressed genes (DEGs) were identified. Functional classification of these DEGs revealed that hexanal down-regulated the expression of genes related to the biosynthesis of ethylene, ABA and cell wall-degrading enzymes, thereby reducing the sensitivity of cells in the fruit AZs to ethylene and ABA [21]. In another transcriptomic study of *Camellia*, the pathways of lignin production, cutin formation, cell wall biogenesis, and ABA response were significantly up-regulated in regulating the shedding of floral organs [22]. In a transcriptomic analysis of abscised and non-abscised small fruits of hickory, a total of 11,976 DEGs were identified, most of the DEGs involved in plant hormone signaling pathways were down-regulated in the abscised small fruits, along with down-regulation of enzymes related to carbohydrate metabolism, indicating that sugar supply of the abscised fruits has been reduced or ceased before they drop [23].

Cotton is an important economic crop and a primary provider of natural fibers, referred to as “white gold” of the textile industry [24]. In recent years, with the development of large-scale cultivation of cotton in China, the planting area of mechanically harvested cotton has rapidly increased, due to its advantages in labor and cost savings compared to manual harvesting [25], accounting for about 50% of the total cotton planting area [26]. However, the low defoliation rate before mechanical harvesting severely restricts the fiber quality of cotton. Therefore, the application of chemical defoliants before harvesting to induce early leaf shedding is a critical issue in improving cotton fiber quality [27]. Consequently, developing cotton varieties that are sensitive to chemical defoliants is a key factor in applying mechanical harvesting and enhancing cotton fiber quality. In this study, two upland cotton cultivars showing diverse genotypes against thidiazuron (TDZ, a chemical defoliant) resistance were chosen to perform RNA sequencing (RNA-seq) analysis under a time-series of treatment (1, 3, and 5 DPT), namely FU75 and 518-48. A large number of DEGs that responded to TDZ treatment were identified through multi pair-comparisons, and then weighted gene co-expression network analysis (WGCNA) was performed to investigate their potential functions. This study aimed to screen key genes and lay the foundation for understanding the molecular mechanisms underlying cotton defoliant action.

## 2. Materials and Methods

### 2.1. Plant Materials and Growth Conditions

The cotton plant materials included the TDZ-sensitive line FU75 and TDZ-insensitive line 518-48, both of which were bred by the Seventh Division Agricultural Science Institute. The experiment was performed in the experimental field (44.44° N, 84.99° E) in Kuitun (Yili Kazak Autonomous Prefecture, Xinjiang, China), where it belongs to continental inland arid climate characterized by long sunshine duration, scarce precipitation, and high evaporation, with an annual frost-free period of 170 days and an average temperature of 11 °C. The soil exhibits the following properties: pH 8.0, total salt content 4.3 g·kg^−1^, total nitrogen 0.89 g·kg^−1^, organic matter 14.5 g·kg^−1^, available phosphorus 52.6 mg·kg^−1^, and available potassium 213 mg·kg^−1^. The two materials were planted in the growing season of May 2024, with experimental layout of randomized complete block design and two biological replicates. The field management was following the local standard practices.

### 2.2. Defoliant Treatment

When the plants of FU75 and 518-48 reached boll-opening stage, a commercial defoliant, a mixture of 400 mg·L^−1^ thidiazuron, 2 mL·L^−1^ 40% ethephon and 2 mL·L^−1^ defoliant adjuvant, together termed TDZ, was applied via spraying. The application was performed in the afternoon under a clear day with a temperature of 20–25 °C, after one week consecutive sunny weather at an application rate of 600 L/hm^2^. The plants treated with water were used as control. The leaf AZs of the second and third young leaves from the top of cotton plants were sampled at 1, 3, and 5 DPT [3], and each sample consisted of a mixture of AZs collected from five plants. The sampling location is the detachment area of petioles where AZs will form, a small segment approximately 3–5 mm long, including both part of stem cortical tissue and part of petiole base tissue. All the samples were immediately frozen in liquid nitrogen and stored in −80 °C until use. A total of 24 samples were made including two lines, three sampling times and two biological replicates.

### 2.3. RNA Sequencing and Analyses

Total RNA was extracted from the leaf AZs using the Spectrum Plant Total RNA Kit (Sigma Aldrich, Saint Luis, MO, USA). RNA quality was assessed using the Agilent Bioanalyzer 2100 system prior to library construction. mRNA was purified from total RNA, and libraries were sequenced on the Illumina HiSeq platform (NEB, Ipswich, MA, USA), generating 150 bp paired-end reads. Low-quality reads, adapter-containing reads, and poly-N-containing reads were removed from the raw reads and then the clean reads were aligned to the cotton genome (*Gossypium hirsutum* TM-1 (AD)_1_). The mapped reads of the identified genes were used for gene expression analysis, and gene expression levels were normalized by FPKM. Subsequently, those data were utilized for DEG identification by the limma package of R software (version 4.0.2) with selection criteria set as a fold change ≥2 and a false discovery rate (FDR) < 0.01, which were in proper oder subjected to sample groups, data trim, data matrix, fit linear module, and DEG confimation [28]. The enrichment analyses of GO and KEGG pathway were performed on the identified DEGs by the online wetsite (http://www.omicshare.com/tools/, accessed on 24 February 2023), among which the signficant threshold was set as an adjuested *p* < 0.05, meanwhile the background gene set was chosen from the annotation infomation of (*G. hirsutum* TM-1 (AD)_1_, http://cotton.zju.edu.cn/, accessed on 1 September 2022) [29].

### 2.4. qRT-PCR Experiment

To verify the accuracy of RNA-seq data, the same RNA samples were subjected to reverse transcription for cDNA synthesis by the HiScript IV 1st Strand cDNA Synthesis Kit (Vazyme, Nanjing, China). Then, the cDNA template was utilized to perform qRT-PCR experiment using the Taq Pro Universal SYBR qPCR Master Mix (Vazyme, Nanjing, China) on a Applied Biosystem^®^ 7500 Fast (Applied Biosystems, Foster City, CA, USA). *GHUBQ7* was chosen as the reference gene for the expression level correction, and the special primers of the selected DEGs were designed by the online-tool of NCBI. The sequences of the primers were listed in Table S2. The qRT-PCR experiment was conducted in 20 μL system under the followed conditions: (1) 1 cycle of 94 °C for 15 s; (2) 40 cycles of 94 °C for 5 s, 60 °C for 30 s; (3) 1 cycle of 60 °C for 45 s. Three technical replicates were performed for calcualting the relative expression levels of those selected DEGs with the algorithm of 2^−ΔΔCt^ [30].

## 3. Results

### 3.1. Quality Evaluation of RNA-Seq Data

**Figure 1:**
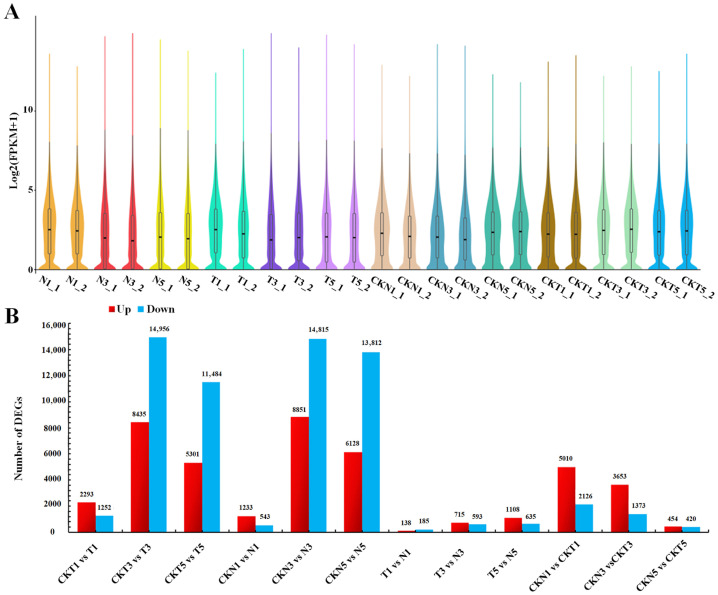
Quality analysis of RNA-seq data and DEGs identification (**A**) Genes expression levels in samples (T and N represent FU75 and 518-48 treated with TDZ, respectively; CKT and CKN represent the control of FU75 and 518-48 treated with water, respectively; T1, T3, and T5 represent treatment durations of 1 day, 3 days, and 5 days, respectively; T1_1 and T1_2 denote the biological replicates). (**B**) DEGs identification from the 12 pair-wise comparisons.

To systematically elucidate the key genes and signaling pathways involved in plant response to TDZ-induced defoliation, RNA-seq was performed on the samples of leaf AZs of cotton lines FU75 and 518-48, which were sampled on 1, 3, 5 days post treatment (DPT) of either water (the control) or TDZ. For convenience, the samples of TDZ-sensitive FU75 and TDZ-resistant 518-48 treated with TDZ were abbreviated as T and N, respectively, while the samples treated with water were abbreviated as CKT and CKN, respectively. Totally, 1,541,914,520 raw reads were obtained from the 24 samples with two biological replicates, retaining 1,505,720,260 clean reads after filtering. The Q30 base percentage was no less than 92.77%, and the GC content was consistently above 43.13% (Table S1). Aligning the RNA-seq data to the annotated reference genome identified 57,116 unique genes, with varying expression levels across samples (Figure 1A), among which, the sample CKN3_1 exhibited the highest gene expression level. Principal component analysis (PCA) of the 24 samples to validate the accuracy of the RNA-seq data resulted in two principal components, PC1 and PC2, which accounted for 24.74% and 11.24% of the total variance, respectively. Biological replicates at different DPT showed high similarity across samples (Figure S1). Besides, Pearson correlation coefficient (PCC) analysis of all RNA-seq samples demonstrated strong correlations in gene expression among samples (Figure S2). These results indicated that the high-quality transcriptomic data meet the standards for subsequent DEG identification and functional enrichment analyses.

### 3.2. Analysis of Differentially Expressed Genes (DEGs)

A total of 35,739 DEGs were identified through 9 pairwise comparisons across all the samples (Supplemental Excel S1). There were more DEGs identified in the pair-wise comparison between TDZ-sensitive and control samples (CKT3-vs-T3, CKN3-vs-N3, CKT5-vs-T5, and CKN5-vs-N5) than in the pair-wise comparison between TDZ-resistant and control samples (CKT1-vs-T1 and CKN1-vs-N1). The number of DEGs exhibited a consistent increasing trend with DPT advances (1, 3, and 5 DPTs). In pair-wise comparisons between different lines at each DPT, fewer DEGs were observed in the pairs of T1-vs-N1, T3-vs-N3, and T5-vs-N5 than in the pairs of CKT1-vs-CKN1, CKT3-vs-CKN3, and CKT5-vs-CKN5. Notably, CKT1-vs-CKN1 showed the highest number of DEGs, while T1-vs-N1 the fewest.

### 3.3. Identification and Functional Enrichment Analyses of DEGs

**Figure 2:**
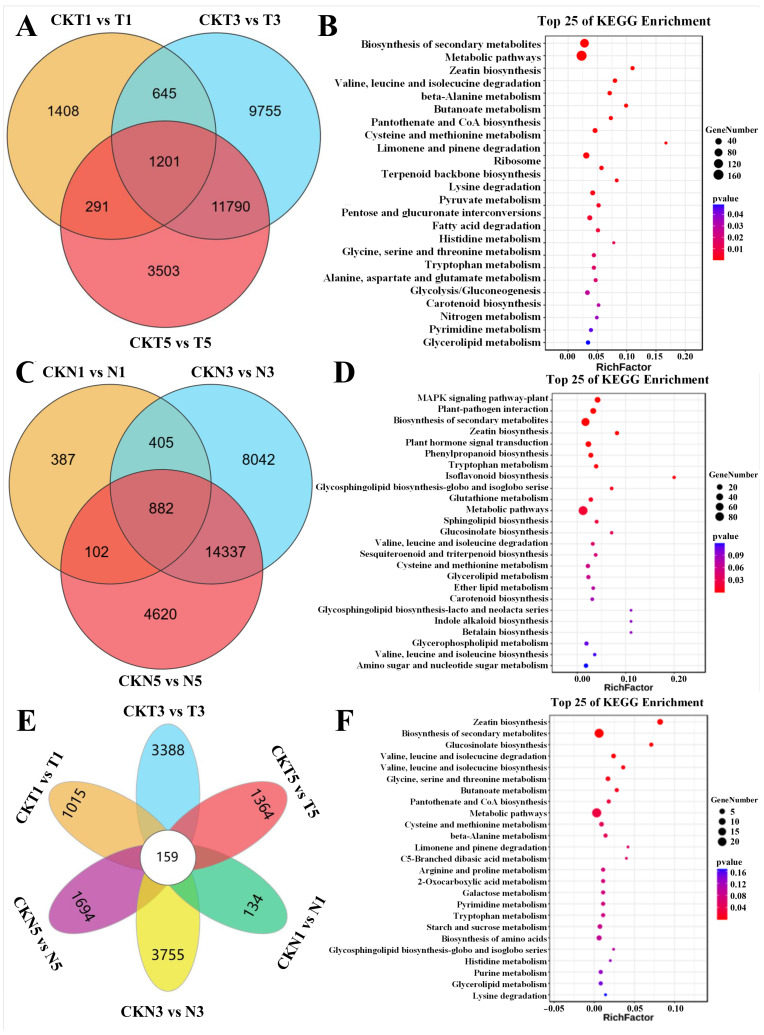
Identification and functional analyses of the specially enriched DEGs. (**A**) Venn diagram and (**B**) KEGG enrichment analysis of 1201 common DEGs in FU75. (**C**) Venn diagram and (**D**) KEGG enrichment analysis of 882 common DEGs in 518-48. (**E**) Venn diagram and (**F**) Enrichment analysis of 159 common DEGs at different time points after TDZ treatment between FU75 and 518-48. T and N represent FU75 and 518-48 treated with TDZ, respectively; CKT and CKN represent the water control groups for FU75 and 518-48, respectively; T1, T3, and T5 represent treatment durations of 1 day, 3 days, and 5 days, respectively.

Of the 1201 DEGs identified in the comparisons between different treatment stages of FU75 at 1, 3, and 5 DPTs of TDZ treatment (Figure 2A), the most enriched KEGG pathways included biosynthesis of secondary metabolites, metabolic pathways, zeatin biosynthesis, valine, leucine, and isoleucine degradation, and beta-Alanine metabolism (Figure 2B). GO enrichment analysis of these 1201 DEGs, showed that the top-5 enriched biological process (BP) terms were regulation of hormone levels (GO:0010817), hormone metabolic process (GO:0042445), cytokinin metabolic process (GO:0009690), cellular hormone metabolic process (GO:0034754), and amine metabolic process (GO:0009308) (Figure S3A).

Similarly, of the 882 DEGs identified in the comparisons between different treatment stages of 518-48 at 1, 3, and 5 DPTs of TDZ treatment (Figure 2C), the most enriched pathways included the MAPK signaling pathway-plant, plant-pathogen interaction, biosynthesis of secondary metabolites, zeatin biosynthesis, and plant hormone signal transduction (Figure 2D). GO enrichment analysis of these 882 DEGs showed that they were significantly enriched in the BP terms of cytokinin metabolic process (GO:0009690), cellular hormone metabolic process (GO:0034754), regulation of hormone levels (GO:0010817), hormone metabolic process (GO:0042445), and multi-organism process (GO:0051704) (Figure S3B).

Of the 159 common DEGs identified from cross-comparison between 518-48 and FU75 at different DPT of TDZ treatment (Figure 2E), the most significantly enriched KEGG pathways were zeatin biosynthesis, biosynthesis of secondary metabolites, glucosinolate biosynthesis, and degradation and biosynthesis of valine, leucine and isoleucine (Figure 2F). GO enrichment analysis of these DEGs showed that they were significantly enriched BP terms of Cytokinin metabolic process (GO:0009690), cellular hormone metabolic process (GO:0034754), regulation of hormone levels (GO:0010817), hormone metabolic process (GO:0042445), and amine metabolic process (GO:0009308) (Figure S3C).

**Figure 3:**
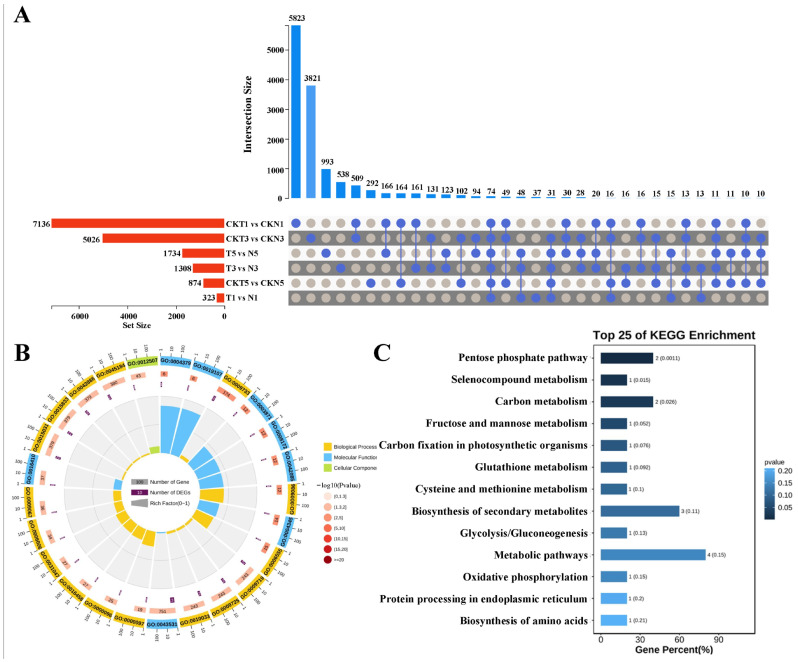
Identification and functional enrichment analysis of the common DEGs across the same material under different periods of TDZ treatment. (**A**) UpSet visualization of the common DEGs among the different samples. (**B**) GO enrichment analysis of the 74 commom DEGs enriched in all the samples. (**C**) KEGG enrichment analysis of the 74 commom DEGs enriched in all the samples. T and N represent FU75 and 518-48 treated with TDZ, respectively; CKT and CKN represent the water control groups for FU75 and 518-48, respectively; T1, T3, and T5 represent treatment durations of 1 day, 3 days, and 5 days, respectively.

Finally, of the 74 DEGs identified from comparison of the two varieties at each of DPTs of TDZ treatment (Figure 3A), GO enrichment analysis showed that they were significantly enriched in the molecular function (MF) terms of glycylpeptide N-tetradecanoyltransferase activity (GO:0004379), myristoyltransferase activity (GO:0019107), and 5-methyltetrahydropteroyltriglutamate-homocysteine S-methyltransferase activity (GO:0003871), the cellular component (CC) terms of ER to Golgi transport vesicle membrane (GO:0012507), COPII vesicle coat (GO:0030127), and ER to Golgi transport vesicle (GO:0030134), and the BP terms of response to auxin (GO:0009733), methionine biosynthetic process (GO:0009086), and methionine metabolic process (GO:0006555) (Figure 3B). The significantly enriched KEGG pathways were pentose phosphate pathway, selenocompound metabolism, carbon metabolism, fructose and mannose metabolism, and carbon fixation in photosynthetic organisms (Figure 3C). To sum up, zeatin biosynthesis, biosynthesis of secondary metabolites, and hormone metabolic process were consistently enriched in comparisons between the two varieties, suggesting their potential critical roles in cotton’s response to TDZ treatment.

### 3.4. Temporal Expression Patterns of DEGs

**Figure 4:**
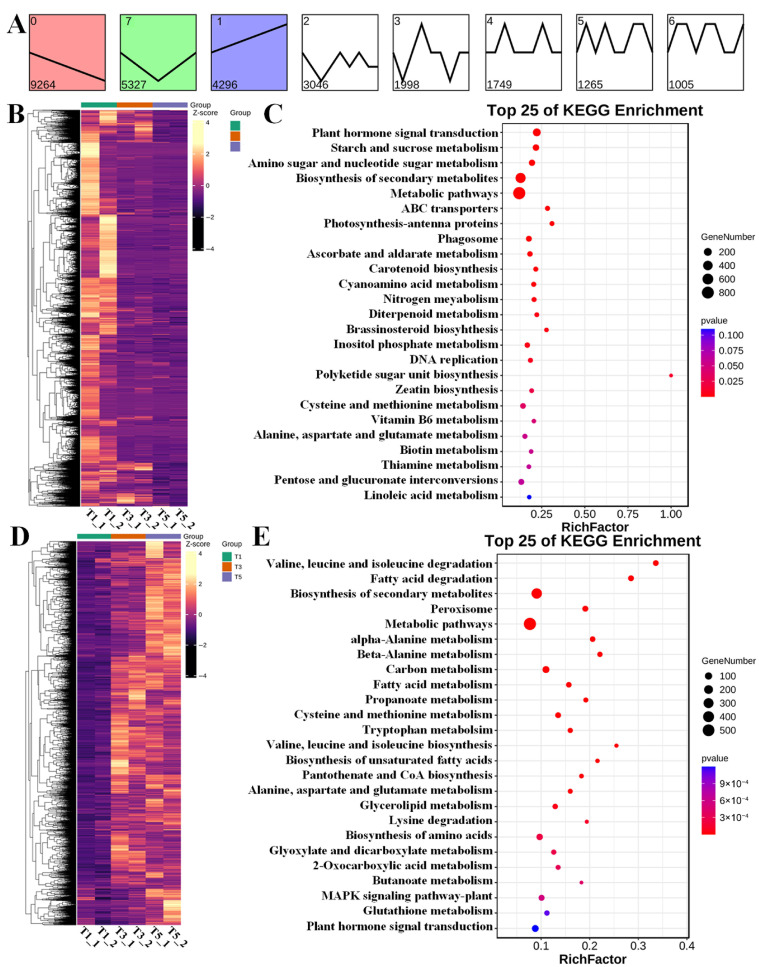
Analyses of expression pattern, clustering, and functional enrichment of DEGs in FU75 samples. (**A**) The results of STEM analysis of DEGs in FU75. (**B**,**C**) The heatmaps and KEGG analysis of DEGs in expression profile 0, respectively. (**D**,**E**) The heatmaps and KEGG analysis of DEGs in expression profile 7, respectively. T represent FU75 treated with TDZ; T1, T3, and T5 represent treatment durations of 1 day, 3 days, and 5 days, respectively; T1_1 and T1_2 denote the two biological replicates.

**Figure 5:**
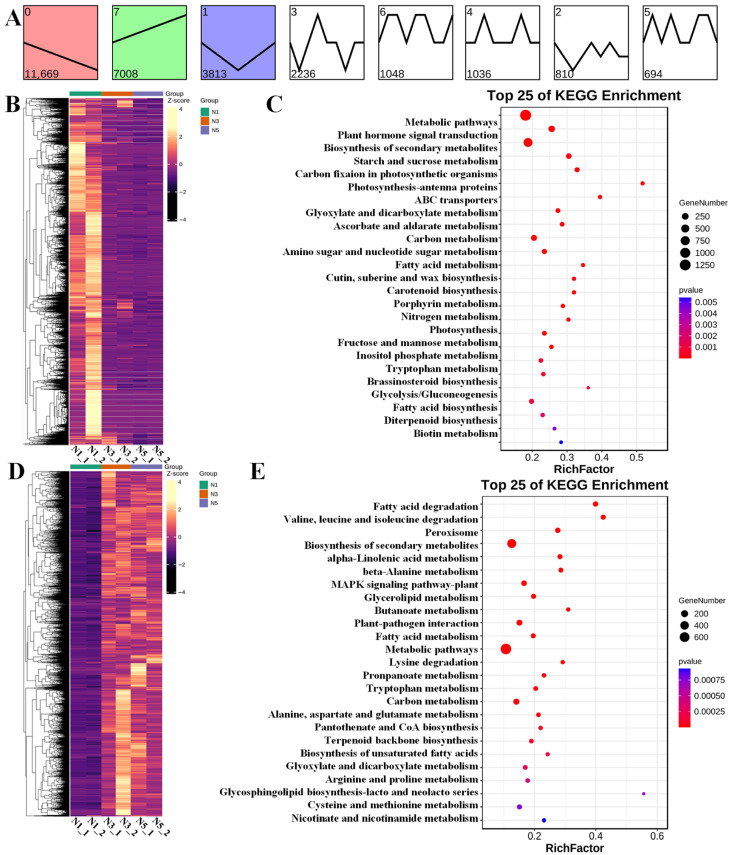
Analyses of expression pattern, clustering, and functional enrichment of DEGs in 518-48 samples. (**A**) The results of STEM analysis of DEGs in FU75. (**B**,**C**) The heatmaps and KEGG analysis of DEGs in expression profile 0, respectively. (**D**,**E**) The heatmaps and KEGG analysis of DEGs in expression profile 7, respectively. N represent 518-48 treated with TDZ; N1, N3, and N5 represent treatment durations of 1 day, 3 days, and 5 days, respectively; N1_1 and N1_2 denote the two biological replicates.

The spatiotemporal expression patterns of the DEGs via short time-series expression miner (STEM) analysis resulted in 28,593 and 28,776 annotated DEGs in FU75 and 518-48, respectively (Figure 4A and Figure 5A). The DEGs in FU75 were significantly enriched into three expression profiles, namely profile 0, profile 7, and profile 1. Profile 0 clustered 9264 DEGs (32.40%) and exhibited a continuous down-regulation trend; profile 1 clustered 4296 DEGs (15.02%) and exhibited an overall up-regulation trend; and profile 7 clustered 5327 DEGs (18.63%) and exhibited an initial down-regulation followed by an up-regulation trend. Similarly, the DEGs in 518-48 (98.40% of the total DEGs) were also clustered into three profiles with similar expressing patterns as in FU75. Profile 0 exhibited a continuous downregulation trend and clustered 11,691 DEGs (40.55%). Profile 7 exhibited a continuous upregulation trend and clustered 7008 DEGs (24.35%). Profile 1 displayed an initial down-regulation followed by a continuous upregulation trend and clustered 3813 DEGs (13.25%).

The DEGs of Profile 0 and Profile 7 in FU75 were separately subjected to clustering analyses, of which the continuous down-regulated and up-regulated expression patterns were consistent with STEM results (Figure 4B,D). KEGG functional analyses of these DEGs revealed that the down-regulated DEGs in Profile 0 were enriched in the pathways of plant hormone signal transduction, starch and sucrose metabolism, amino sugar and nucleotide sugar metabolism, biosynthesis of secondary metabolites, and metabolic pathways (Figure 4C). The top-5 KEGG pathways of the up-regulated DEGs in Profile 0 were valine, leucine and isoleucine degradation, fatty acid degradation, biosynthesis of secondary metabolites, peroxisome, and metabolic pathways (Figure 4E).

Likewise, functional enrichment analyses of the DEGs of Profile 0 and Profile 7 in 518-48 showed continuous down-regulation and up-regulation trends (Figure 5B,D), respectively, both of which displayed the high consistencies with their STEM results. In Profile 0, the most significantly enriched KEGG pathways included metabolic pathways, plant hormone signal transduction, biosynthesis of secondary metabolites, starch and sucrose metabolism, and carbon fixation in photosynthetic organisms (Figure 5C). The DEGs in Profile 7 were significantly enriched in the KEGG pathways of fatty acid degradation, valine, leucine and isoleucine degradation, peroxisome, biosynthesis of secondary metabolites, and alpha-linolenic acid metabolism (Figure 5E).

### 3.5. WGCNA of TDZ Treatment-Related DEGs

**Figure 6:**
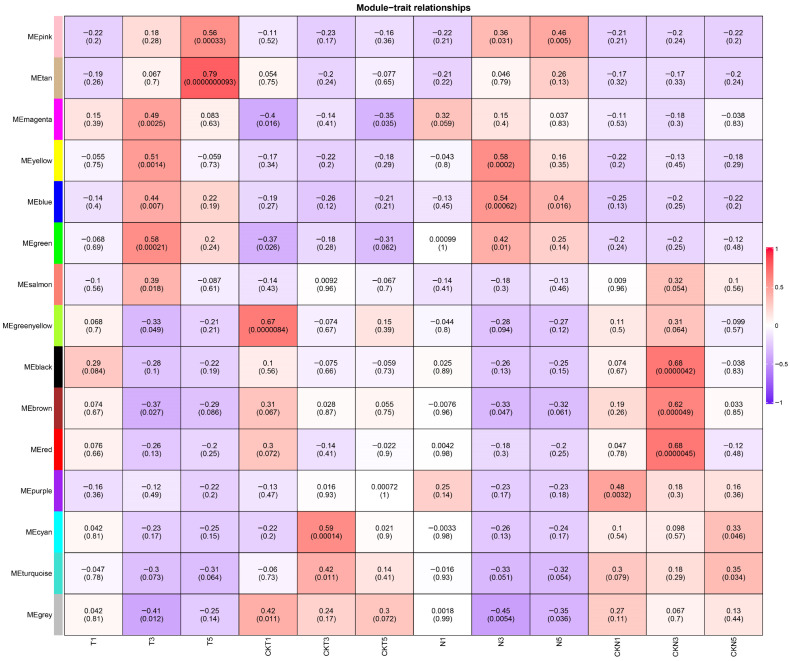
Module−trait relationships analysis of DEGs in all the samples. T and N represent FU75 and 518-48 treated with TDZ, respectively; CKT and CKN represent the water control groups for FU75 and 518-48, respectively; T1, T3, and T5 represent treatment durations of 1 day, 3 days, and 5 days, respectively.

To screen the potentially hub genes that might make significant contributions to the response of plants to TDZ treatment, WGCNA was performed on the expression matrix of cotton samples treated with TDZ after systematically excluding data from outlier samples (Figure S4A). The optimal soft threshold of 29 was selected for the analysis based on the scale-free topology criterion (Figure S4B). All co-expression modules were distinctly identified and assigned unique color codes, with the grey module representing genes that were considered unassigned or excluded from clustering. The interrelationships among these modules were further visualized using a heatmap (Figure S4C). Through consensus module-trait association analysis, gene set T5 (MEtan module, comprising 45 differentially expressed genes) was found to be significantly correlated with TDZ treatment (Figure 6). The relationship between module membership and gene significance within the MEtan module is presented in scatter plots (Figure S4D).

**Figure 7:**
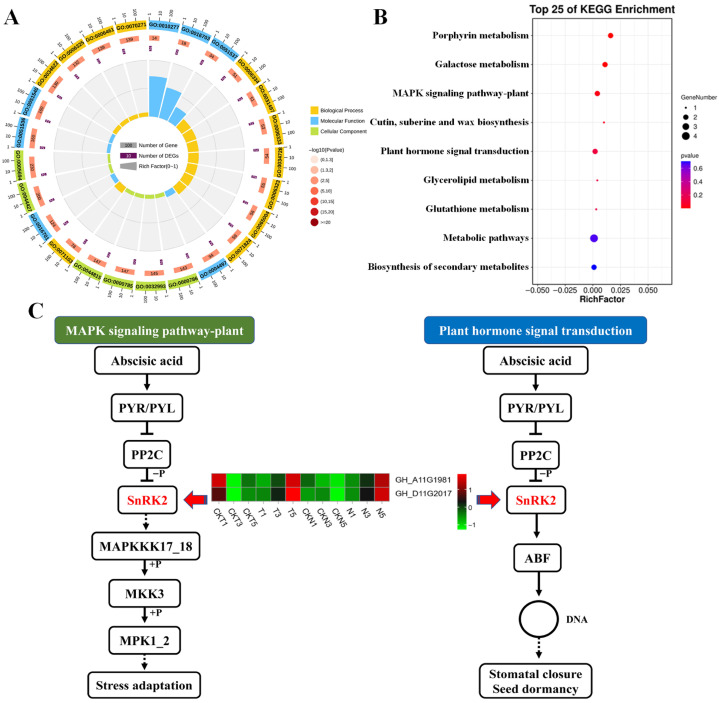
Functional enrichment analyses and signaling pathways of gene set T5 (MEtan module, 45 genes). (**A**,**B**) GO and KEGG enrichment analysis of gene set T5 (MEtan module, 45 genes). (**C**) Construction of the MAPK signaling pathway-plant and plant hormone signaling transduction pathways. T and N represent FU75 and 518-48 treated with TDZ, respectively; CKT and CKN represent the water control groups for FU75 and 518-48, respectively; T1, T3, and T5 represent treatment durations of 1 day, 3 days, and 5 days, respectively.

GO functional enrichment analysis of these 45 DEGs (Figure 7A) resulted in the most significantly enriched MF terms of chlorophyllide a oxygenase [overall] activity (GO:0010277), oxidoreductase activity, acting on single donors with incorporation of molecular oxygen, incorporation of one atom of oxygen (internal monooxygenases or internal mixed function oxidases) (GO:0016703), and 2 iron, 2 sulfur cluster binding (GO:0051537), and BP terms of nucleosome assembly (GO:0006334), chromatin assembly (GO:0031497), and the CC terms of chromatin assembly or disassembly (GO:0006333), and the nucleosome (GO:0000786), protein-DNA complex (GO:0032993), and chromatin (GO:0000785). KEGG functional analysis of these DEGs resulted that they were significantly enriched in the pathways of porphyrin metabolism, galactose metabolism, MAPK signaling pathway-plant, cutin, subersine and wax biosynthesis, and plant hormone signal transduction (Figure 7B). Based on the above-mentioned KEGG pathway analysis of DEGs screened through different comparisons, it was noticed that plant hormone signal transduction and the MAPK signaling pathway-plant were repeatedly enriched, implying their potential contributions to the response of cotton plants to TDZ treatment. Interestingly, abscisic acid (ABA), as endogenous hormone that actively participate in multiple biological processes (such as growth and development, or adversity stresses), was often found to play a key role in plant hormone signal transduction and MAPK signaling pathways. A core gene *sucrose non-fermenting 1 (SNF1)-related protein kinase 2* (*SnRK2*, *GH_D11G2017* and *GH_A11G1981*) in this pathway was selected to perform heatmap analysis (Figure 7C). In all the control samples (CKT1-CKT5 and CKN1-CKN5), both of the two *SnRK2* genes showed down-regulated expression patterns, while, their expression levels were continuously up-regulated in the samples of T1-T5 and N1-N5, reaching a maximum value at 5 DPA. These results indicated that ABA signal pathway might be involved in cotton plant adapting to adverse stresses through activating the core gene *SnRK2*.

### 3.6. Verification of Gene Expression by qRT-PCR

**Figure 8:**
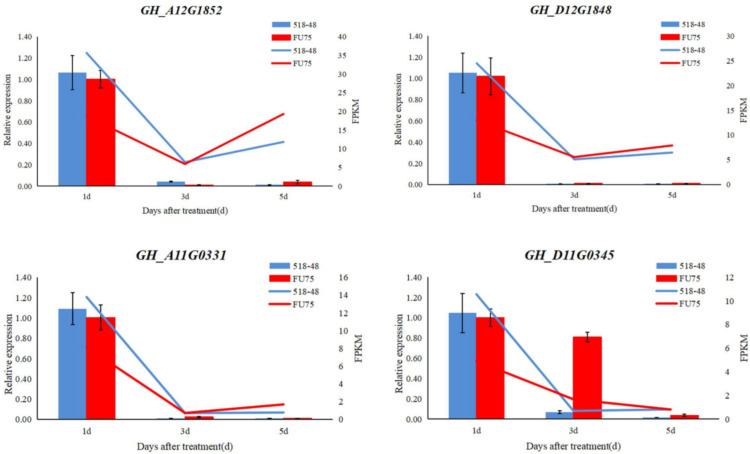
The qRT-PCR Results conducted on the randomly chosen DEG. The *X*-axis on the left represents the relative expression of qRT-PCR, and the *X*-axis on the right represents the PFKM vales of RNA-seq data. The blue columnar and lines represent the relative expression of qRT-PCR and PFKM vales of RNA-seq data in 518-48, and the red columnar and lines represent the the relative expression of qRT-PCR and PFKM vales of RNA-seq data in FU75.

To confirm the accuracy and reliability of our RNA-seq data, 4 DEGs were randomly selected to perform qRT-PCR experiment. The qRT-PCR results (the bar chart) showed that the expression trends of the four DEGs were highly consistent with the transcriptome results (the line graph) (Figure 8). These results were consistent with RNA-seq data.

## 4. Discussion

As a major source of natural fiber, cotton plays an important role in agriculture and textile industry, providing substantial economic benefits. Mechanical harvesting has been successfully applied in cotton fiber picking in developed countries. In recent years, due to the increasing of labor costs, mechanical harvesting has gradually replaced manual harvesting in developing countries. However, the retained leaves on cotton plants is one of the key factors affecting mechanical harvesting due to its impairing on the quality of cotton fibers. Therefore, promoting leaf abscission with chemical defoliant before harvesting not only allows more nutrients to be transferred to the bolls but also improves the quality of cotton fibers. However, the molecular mechanism of cotton plant response to defoliant is still not clear. In a previous study of applying TDZ to cotton plants, it was observed that after treated with TDZ at eight true leaf stages of cotton plants (CCRI 49 and CCRI 12), the contents of reactive oxygen species (ROS) and malondialdehyde (MDA) were significantly increased, photosynthetic efficiency and photosynthesis-related genes were significantly decreased, and TDZ application significantly enhanced AZ formation and leaf shedding [4]. In addition, transcriptomic sequencing and enzyme-linked immunosorbent assay (ELISA) analysis demonstrated that TDZ treatment significantly reduced the expression of cytokinins and auxins in leaves. A KEGG analysis of DEGs after TDZ treatment indicated that the synthesis, metabolism, and signal transduction of auxins, cytokinins, and brassinosteroids might be involved in TDZ-induced cotton leaf abscission [31,32], while the effect of Ethylene treatment did not show significant changes [33]. These studies demonstrated that TDZ can effectively promote cotton leaf abscission. It is of great practical significance for agricultural development to control leaf abscission via understanding it genetic mechanisms and identifying key genes regulating leaf abscission, and for developing new cotton varieties adaptive to mechanical harvesting [34].

Currently, RNA-seq has been widely applied to elucidate the molecular genetic mechanisms underlying organ or tissue abscission in plants, such as in litchi [35], rice [36], soybean [37], and tomato [38]. In this study, a total of 35,739 DEGs were identified through pairwise comparisons of transcriptomic data across different TDZ treatment samples. These DEGs were mainly enriched in pathways of zeatin biosynthesis, secondary metabolite biosynthesis, and hormone metabolic processes. Integrated transcriptomic and miRNA-seq analyses of Korla fragrant pear plants revealed that the calyx-persistent plants exhibited significant enrichment in zeatin biosynthesis, plant hormone signal transduction, and carotenoid biosynthesis pathways compared to the calyx-abscission ones [39]. These findings suggested that zeatin biosynthesis might make great contributions to cotton response to TDZ-induced abscission.

An important observation in this study is that key pathways such as biosynthesis of secondary metabolites, peroxisome, and alpha-linolenic acid metabolism are highly correlated with leaf abscission. Previous study of a comparative transcriptomic analysis of two lemon varieties showed that a higher number of DEGs were identified during the mid-abscission stage, with enrichment in alpha-linolenic acid metabolism [40]. Peroxidases can catalyze and oxidize lignin formation, regulating lignin structure to facilitate precise cell separation and abscission [41]. Secondary metabolites such as flavonoids are known to regulate plant organ abscission [42], while phenolic compounds can counteract the harmful effects of reactive oxygen species, reducing boll abscission and improving yield [43]. These findings are highly consistent with our transcriptomic results, underscoring the critical role of these pathways in regulating cotton leaf abscission.

Studies have shown that ABA can promote organ abscission and senescence in plants [44]. In mulberry, MaABF1 (ABA-binding factor/ABA-responsive element-binding protein) promotes fruit abscission by regulating the expression of MaJOINTLESS [45]. In Arabidopsis, CDF4 positively regulates leaf senescence by modulating the ABA pathway [46]. ABA also plays a significant role in organ or tissue abscission in plants such as sweet cherry [47], apple [25], *Salvia miltiorrhiza* [48], and pistachio [49]. The MAPK cascade reaction also plays a pivotal role in regulating plant organ abscission. In litchi, LcMPK6 and LcMPK3 positively regulate small fruit abscission [50]. In *Arabidopsis*, the MAPK cascade exerts redundant functions at multiple developmental stages and regulates floral organ abscission [51]. This helps us understand the molecular mechanisms of plant hormone signal transduction and the MAPK signaling pathway in cotton leaf abscission. This study constructed the plant hormone signal transduction and MAPK signaling pathway and identified a key gene, *SnRK2*, shared between the two pathways. SnRK is a class of Ser/Thr protein kinases widely present in plants and plays an important role in various plant stresses. Based on domain differences, they are divided into three subfamilies (SnRK1, SnRK2, and SnRK3), among which SnRK2 plays a significant role in abiotic stress and ABA signal transduction [52,53]. For example, in the genome-wide analysis of pepper, SnRK2 plays a crucial role in fruit development [54]. In strawberry, increased ABA content inhibits SnRK2.6, thereby phosphorylating bHLH3 to promote fruit ripening and coloration [55]. Therefore, *SnRK2* could be chosen as a crucial candidate gene to perform the further functional verification, of which the transgenic lines will be practically applied for cotton leaf abscission in the future.

## 5. Conclusions

Large-scale mechanization is the inevitable trend of modern agriculture, and the utilization of crop defoliants will greatly improve the efficiency of agricultural production. To dissect the molecular mechanism of cotton leaf abscission, RNA-seq analysis was performed on the AZ samples of TDZ-sensitive cultivar FU75 and TDZ-resistant cultivar 518-48 after TDZ treatment, and a plenty of DEGs related to TDZ treatment were identified via multiple pair comparisons. Common DEGs identification, GO and KEGG enrichment analyses of these common DEGs indicated that zeatin biosynthesis, biosynthesis of secondary metabolites, and hormone metabolic process might positively regulate cotton response to TDZ treatment. STEM and WGCNA analyses of these DEGs demonstrated that SnRK2 was the candidate gene in this dataset. These results provide new insights into the molecular mechanisms of cotton defoliants and improving fiber quality. However, further studies, including transgenic modification and gene editing, are still needed to validate the gene function and to dissect its molecular mechanism in regulating leaf abscission induced by chemical defoliant. Once validated and integrated with phenotypic metrics, these candidate genes could aid defoliation-related breeding and cotton production.

## Data Availability

The raw data reported in this paper have been deposited and are available in the Genome Sequence Archive in National Genomics Data Center, China National Center for Bioinformation/Beijing Institute of Genomics, Chinese Academy of Sciences (GSA: CRA025766) that are publicly accessible at https://ngdc.cncb.ac.cn/gsa (accessed on 10 August 2021).

## Supplementary Materials

The following supporting information can be downloaded at: https://www.mdpi.com/article/10.3390/biology15010074/s1, Figure S1: Principal component analysis of the 24 samples; Figure S2: Pearson correlation coefficient analysis of the 24 samples; Figure S3: GO enrichment analyses of the differently enriched common DEGs. (A) GO enrichment analysis of the common 1201 DEGs among FU75 samples at 1, 3, and 5 DPTs. (B) GO enrichment analysis of the common 882 DEGs among 518-48 samples at 1, 3, and 5 DPTs. (C) GO enrichment analysis of the common 159 DEGs between FU and 518-48 samples at different DPT; Figure S4: WGCNA of DEGs in all the 24 smaples. (A) The expression matrix of cotton samples treated with TDZ. (B) The scale-free topology criterion in WGCNA results. (C) The relationships of different modules in a heatmap. (D) The association beyween module membership and gene significance; Table S1: Analysis of Cotton RNA-seq under TDZ Treatment and contorl; Table S2: Primer design for the randomly selected genes; Excel S1: The information of expressed gene and all the DEGs or the specific DEGs.
