# Supplementary material for: Transcriptional Regulation and WGCNA Studies of Leaf Abscission in Cotton Cultivars FU75 and 518-48 Under Chemical Defoliant Treatment
Source: Biology (Basel). 2025 Dec 31;15(1):74. doi: 10.3390/biology15010074 (PMC12785033; doi:10.3390/biology15010074)
Supplement: Supplementary file 1 [file biology-15-00074-s001.zip › biology-3956358-supplementary/Figure S1-4.docx]

Figure S1: Principal component analysis of the 24 samples





Figure S2: Pearson correlation coefficient analysis of the 24 samples





Figure S3: GO enrichment analyses of the differently enriched common DEGs. (A) GO enrichment analysis of the common 1201 DEGs among FU75 samples at 1, 3, and 5 DPTs. (B) GO enrichment analysis of the common 882 DEGs among 518-48 samples at 1, 3, and 5 DPTs. (C) GO enrichment analysis of the common 159 DEGs between FU and 518-48 samples at different DPT





Figure S4: WGCNA of DEGs in all the 24 smaples. (A) The expression matrix of cotton samples treated with TDZ. (B) The scale-free topology criterion in WGCNA results. (C) The relationships of different modules in a heatmap. (D) The association beyween module membership and gene significance;
