# Supplementary material for: Transcriptional Regulation and WGCNA Studies of Leaf Abscission in Cotton Cultivars FU75 and 518-48 Under Chemical Defoliant Treatment
Source: Biology (Basel). 2025 Dec 31;15(1):74. doi: 10.3390/biology15010074 (PMC12785033; doi:10.3390/biology15010074)
Supplement: Supplementary file 1 [file biology-15-00074-s001.zip › biology-3956358-supplementary/Table S1.docx]

Table S1 Analysis of Cotton RNA-seq under TDZ Treatment and control

| Sample | Clean reads | Clean bases | Q30(%) | GC content (%) |
| --- | --- | --- | --- | --- |
| T1_1 | 40869054 | 40048584 | 94.35 | 43.5 |
| T1_2 | 44612854 | 43761234 | 93.21 | 43.47 |
| T3_1 | 43417416 | 42495246 | 93.9 | 43.51 |
| T3_2 | 42072060 | 41097424 | 93.15 | 43.56 |
| T5_1 | 43687100 | 42496016 | 94.31 | 43.52 |
| T5_2 | 41868648 | 40904654 | 94.05 | 43.51 |
| CKT1_1 | 43262500 | 42209936 | 94.06 | 43.66 |
| CKT1_2 | 41080642 | 40315316 | 94.05 | 43.63 |
| CKT3_1 | 42456088 | 40914390 | 93.24 | 43.79 |
| CKT3_2 | 43817078 | 43280228 | 94.09 | 43.21 |
| CKT5_1 | 39616026 | 38745686 | 94.36 | 43.42 |
| CKT5_2 | 42184130 | 41249822 | 93.9 | 43.49 |
| N1_1 | 42040068 | 41213636 | 93.37 | 43.39 |
| N1_2 | 39942218 | 39209904 | 93.76 | 43.7 |
| N3_1 | 43882104 | 42604742 | 93.87 | 43.57 |
| N3_2 | 45093674 | 44088060 | 93.83 | 43.53 |
| N5_1 | 39344334 | 38120720 | 94.21 | 43.72 |
| N5_2 | 40892600 | 40099906 | 92.99 | 43.13 |
| CKN1_1 | 42616868 | 42016058 | 93.53 | 43.67 |
| CKN1_2 | 41367300 | 40087238 | 93.66 | 43.36 |
| CKN3_1 | 42542680 | 41633586 | 93.35 | 43.94 |
| CKN3_2 | 43759548 | 42748076 | 93.24 | 43.85 |
| CKN5_1 | 43374792 | 42350120 | 93.48 | 43.55 |
| CKN5_2 | 46994466 | 45709804 | 93.39 | 43.56 |
| Average | 42533095 | 41558349 | 93.72 | 43.55 |

**Note:** T and N represent FU75 and 518-48 treated with TDZ, respectively, while CKT and CKN represent the control groups of FU75 and 518-48 treated with clear water. T1, T3, and T5 denote treatments for 1 day, 3 days, and 5 days, respectively. T1_1 and T1_2 indicate two replicates. Q30 refers to the percentage of nucleotides with a quality value of ≥ 30. GC content refers to the percentage of cytosine and guanine in high-quality usable reads.
