# Supplementary material for: Transcriptional Regulation and WGCNA Studies of Leaf Abscission in Cotton Cultivars FU75 and 518-48 Under Chemical Defoliant Treatment
Source: Biology (Basel). 2025 Dec 31;15(1):74. doi: 10.3390/biology15010074 (PMC12785033; doi:10.3390/biology15010074)
Supplement: Supplementary file 1 [file biology-15-00074-s001.zip › biology-3956358-supplementary/Table S2.docx]

| **Table S2 Primer design for the randomly selected genes** | | |
| --- | --- | --- |
| **ID** | **Primer** | **5' to 3'** |
| GH_D12G1848 | 1-F | CAAATCCCAGCAACCAAGGC |
|  | 1-R | AATGCCCGTACCCCATTGTT |
| GH_A12G1852 | 2-F | CCGGCAACGTTTTGGACAAT |
|  | 2-R | ACCCAATTGTTGTCCGGGTT |
| GH_D11G0345 | 3-F | TTCGGTCCTCGGGTTTTAGC |
|  | 3-R | TCGGGTCTTCGTTTCCTTCC |
| GH_A11G0331 | 4-F | CGGTTCAAGCATGTCGGTTC |
|  | 4-R | CGTCGGCGTCGTTTCAAATC |
| GHUBQ7 | 5-F | GAAGGCATTCCACCTGACCAAC |
|  | 5-R | CTTGACCTTCTTCTTCTTGTGCTTG |
